# ARTT Approach to Total Elbow Arthroplasty Devised for Post-Trauma Patients: Preliminary Results

**DOI:** 10.3390/jcm14092901

**Published:** 2025-04-23

**Authors:** Biagio Abate, Martina Coppola, Giuseppe Bardellini, Federico Martinelli, Andrea Celli, Luigi Celli

**Affiliations:** Shoulder and Elbow Unit, Department of Orthopaedic Surgery, Hesperia Hospital, Via Arqua 80, 41125 Modena, Italy; biagioabate.6@gmail.com (B.A.); martinacoppola_96@virgilio.it (M.C.); giuseppebardellini@outlook.it (G.B.); fede.mart@hotmail.it (F.M.); studioprofcelli@gmail.com (L.C.)

**Keywords:** elbow, triceps exposure, fracture malunion, elbow replacement, Anconeus-reflected triceps tongue approach, post-traumatic elbow joint deformity, component malposition

## Abstract

**Background:** An increasing number of total elbow arthroplasty (TEA) procedures are performed in trauma patients every year through a variety of approaches. We have devised the Anconeus-reflected Triceps tongue (ARTT) approach for TEA, which optimizes soft tissue management and implant placement, particularly in post-trauma patients, where extensive scar tissue and/or marked bone deformity hamper joint exposure and carry a risk of component malposition. We describe the ARTT surgical technique, discuss its advantages, and report its preliminary results. **Methods:** Six consecutive patients with malunion of the articular elbow surfaces with severe soft tissue retraction and multiple previous surgeries underwent TEA using the ARTT approach, which spares the triceps tendon insertion on the olecranon and reflects the anconeus and triceps muscles as one. **Results:** At a mean follow-up of 29 months, the Mayo Elbow Performance Score had increased from 39 to 95 points, whereas the visual analog score for pain had fallen from 7.5 to 1. None of the patients had insufficiency or secondary detachment of the triceps tendon and all achieved grade 4 or 5 on the Medical Research Council scale. **Discussion:** The ARTT approach provides enhanced joint exposure, resulting in the preservation of the triceps tendon insertion on the olecranon and enabling earlier active rehabilitation. Our preliminary results indicate that it is a viable alternative to traditional techniques, particularly in post-trauma patients with severe elbow dysfunction, who often suffer from extensive scarring, soft tissue damage, and bone deformity.

## 1. Introduction

Undergoing total elbow arthroplasty (TEA) is a major decision for patients with severe elbow dysfunction, particularly those with post-traumatic arthritis. In such cases, the technical challenges are compounded by distorted anatomy, extensive scarring, and soft tissue damage due to prior surgical procedures or the sequelae of trauma [1,2]. Successful TEA demands not only accurate implant placement but also careful soft tissue management, particularly the extensor mechanism, to ensure functional recovery and durability of the repair [1,2]. The triceps muscle and its attachment to the olecranon are critical for active elbow extension, which underpins most daily activities [3,4,5,6]. Notably, TEA procedures must strike a balance between adequate joint exposure and minimization of triceps damage. In post-trauma patients, the sequelae of previous surgery may require triceps tendon retreatment, which hampers joint exposure and heightens the risk of component malposition [2,3,7,8,9,10]. Poor triceps reconstruction can lead to further complications, including extensor weakness and secondary detachment of the tendon from the olecranon [3,11]. Approaches that prioritize extensor mechanism preservation, such as triceps-sparing techniques, often provide insufficient joint exposure [1,12,13,14,15,16], adversely affecting implant placement and the management of complex deformities and tissue scarring in post-trauma patients [5,6]. In contrast, techniques that prioritize exposure, such as triceps-reflecting approaches, involve detachment of the triceps tendon from its olecranon insertion [5,15,16,17,18,19,20,21]. Although these approaches afford a greater joint visualization, they frequently result in postoperative extensor weakness or even structural failure of the tendon reattachment to the olecranon [3,18,19,22,23,24]. To address these problems, we have devised the Anconeus-reflected Triceps tongue (ARTT) approach, which combines improved joint exposure and triceps tendon management. Reflecting the anconeus and triceps muscle as a single flap affords ample visualization of the joint while preserving the integrity of the triceps tendon attachment to the olecranon. We present the ARTT approach, describe its technical features, and report its preliminary clinical results in patients with complex post-traumatic injury. The study was approved by the Institutional Review Board (0020GHCIRB) and carried out in accordance with the ethical standards of the 1964 Declaration of Helsinki as updated in 2004.

## 2. Materials and Methods

The primary diagnosis of all patients was post-traumatic arthritis secondary to open reduction and internal fixation of intra-articular distal humerus fractures and treatment of the resulting stiff elbow. The mean number of prior procedures was 2.5 (range, 2–4). All patients’ surgical records were carefully examined. In all elbows, the triceps tendon was not detached from the olecranon footprint. Severe stiffness hampered the preoperative evaluation of the extensor mechanism.

A linked semiconstrained Coonrad-Morrey (Zimmer-Biomet, Warsaw, IN, USA) implant was used in all patients, two men and four women whose mean age at the time of the procedure was 67.7 years (range, 57–79). There were two left and four right implants. The patients’ data are reported in Table 1.

All patients were available for clinical and radiographic assessments up to 24 months after the procedure. Pain was determined on a 0–10 visual analog scale (VAS) [25] before the procedure and at the last follow-up. The range of motion (ROM) was measured with a goniometer. We calculated the Mayo Elbow Performance Score (MEPS) based on the clinical data [17].

The extensor mechanism was assessed using the Medical Research Council (MRC) scale as modified by Paternostro-Sluga [26,27]. We also tested the patient’s ability to hold the elbow against gravity with the forearm extended above the head.

### 2.1. Radiographic Assessments

The most recent anteroposterior and lateral x-rays of the elbow were assessed to evaluate any radiolucent lines (RL) around the components and periarticular heterotopic ossification. The RL was subdivided into type 0, no RL or RL involving <1 mm and <50% of interface; type 1, RL involving >1 mm and <50% of interface; type 2, RL involving >1 mm and >50% of interface; type 3, RL involving >2 mm and the whole interface; and type 4, radiographic signs of component loosening [17].

The classification of heterotopic ossification was in line with Hastings and Graham [28]. Accordingly, class I lesions were subclinical; class IIA lesions limited flexion extension; class IIB lesions limited pronation–supination; class IIC lesions limited motion in both planes, and class III lesions equaled complete ankylosis.

### 2.2. Triceps Function Assessment

The original MRC scale does not take into consideration two important parameters: the ROM of a movement and the amount of resistance against which it can be performed [26]. The scale as modified by Paternostro-Sluga [27] addresses this shortcoming by rating the muscle strength related to the potential passive and active ROM. Accordingly, 0 = no muscle contraction; 1 = presence of contraction; 2 = ability to perform a movement actively without gravity; 2 to 3 = active movement against gravity without resistance <50% of feasible ROM; 3 = movement against gravity without resistance >50% of feasible passive ROM; 3 to 4 = movement against light resistance over <50% of feasible passive ROM; 4 = movement >50% of ROM, weaker than contralateral side; 5 = movement against resistance over entire ROM, same resistance as contralateral side.

### 2.3. Surgical Technique

#### 2.3.1. Patient Positioning and Anesthesia

The patient lies supine with the injured arm draped freely. General anesthesia combined with a regional nerve block is used for intraoperative and postoperative analgesia. The elbow is on the chest and a sterile tourniquet was used.

#### 2.3.2. Incision and Exposure

**Figure 1:**
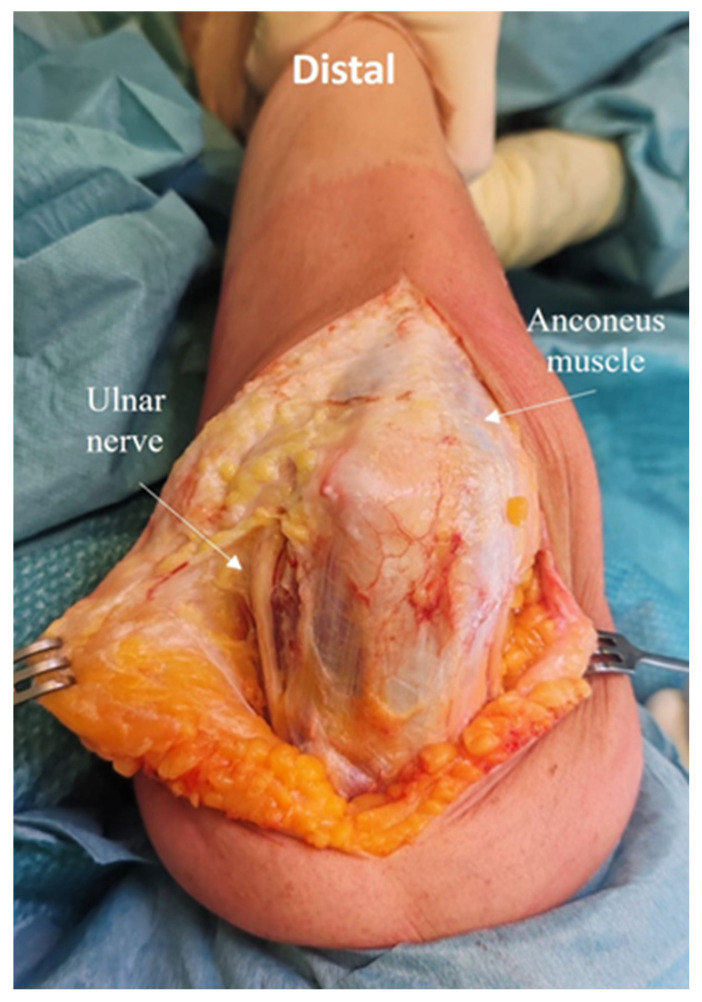
After performing a straight posterior skin incision (15–20 cm), large, thick medial and lateral skin flaps are elevated, and subcutaneous dissection is performed. The ulnar nerve is identified and mobilized, the intermuscular septum is removed.

After performing a straight posterior skin incision (15–20 cm), large, thick medial, and lateral skin flaps are elevated and careful subcutaneous dissection is performed to preserve the posterior antebrachial cutaneous nerve branches, to minimize the risk of postoperative sensory disturbances. The ulnar nerve is identified and mobilized, the intermuscular septum is removed (Figure 1).

### 2.4. Development of the ARTT Approach

Step 1: Creation of the Composite Anconeus-reflected Triceps tongue

**Figure 2:**
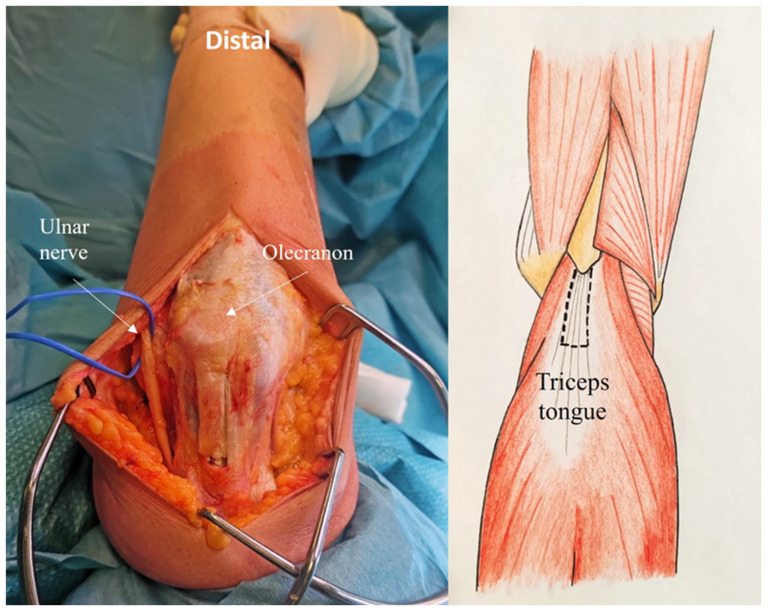
The triceps tongue is a distally based rectangular flap of the triceps tendon, measuring approximately 8 cm in length and 2–3 cm in width.

The superficial tendon of the triceps extensor is visible, the medial portion being thicker than the lateral portion, which is continuous with the anconeus muscle. The tongue approach is based on the anatomical characteristics of the triceps tendon. In fact, the deep muscular portion of the triceps has a tendon insertion originating from the medial head and a superficial tendon insertion originating from the long and lateral heads. These are the anatomical features underpinning the ARTT approach. The triceps tongue is a distally based rectangular flap of the triceps tendon, measuring approximately 8 cm in length by 2–3 cm in width (Figure 2)

**Figure 3:**
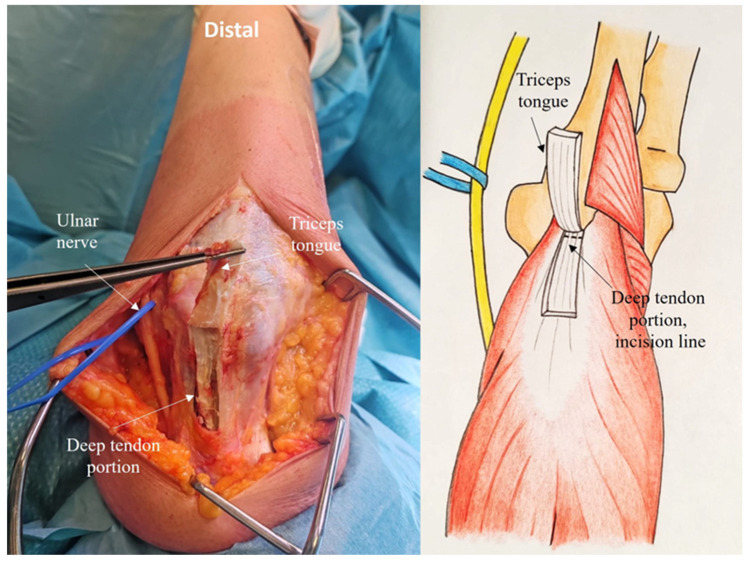
The tendinous portion is elevated, preserving its attachment to the olecranon insertion and leaving part of the deep tendon.

The tendinous portion is elevated, carefully preserving its olecranon insertion and leaving part of the deep tendon (proper tendon of the triceps) in continuity with the underlying muscle (Figure 3).

**Figure 4:**
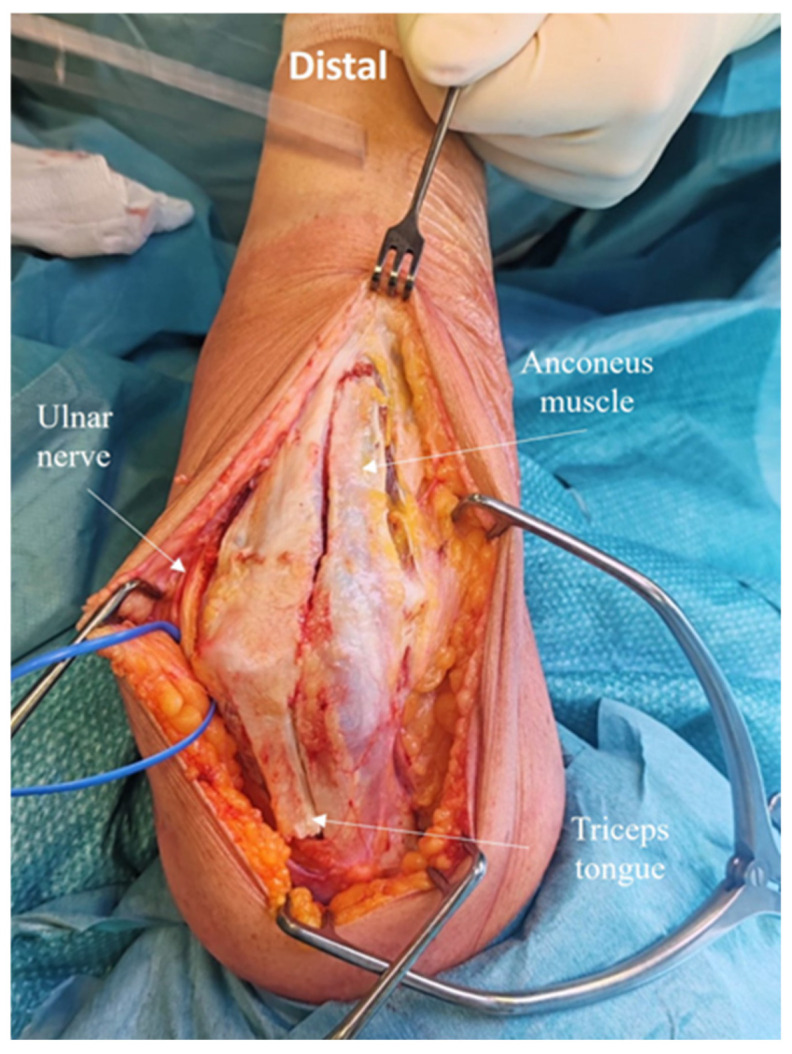
The anconeus muscle is elevated from the ulna, preserving the lateral collateral ligament insertion. A subperiosteal dissection is performed along the radial margin of the ulnar crest.

Laterally, in the Kocher interval, the anconeus muscle is elevated from the ulna, preserving the lateral collateral ligament insertion (Figure 4).

**Figure 5:**
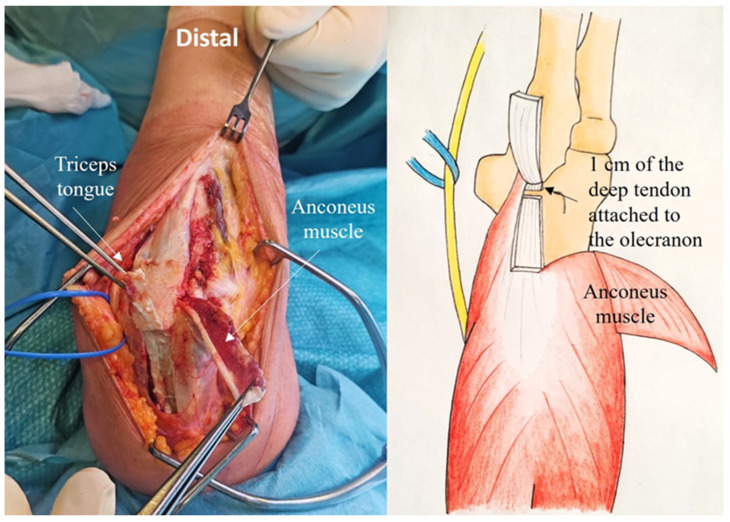
The anconeus muscle is lifted, carefully avoiding damage to its vascular and nerve supplies.

A subperiosteal dissection is performed along the radial margin of the ulnar crest. The anconeus muscle is lifted, avoiding disruption of its vascular and nerve supply [7] (Figure 5).

**Figure 6:**
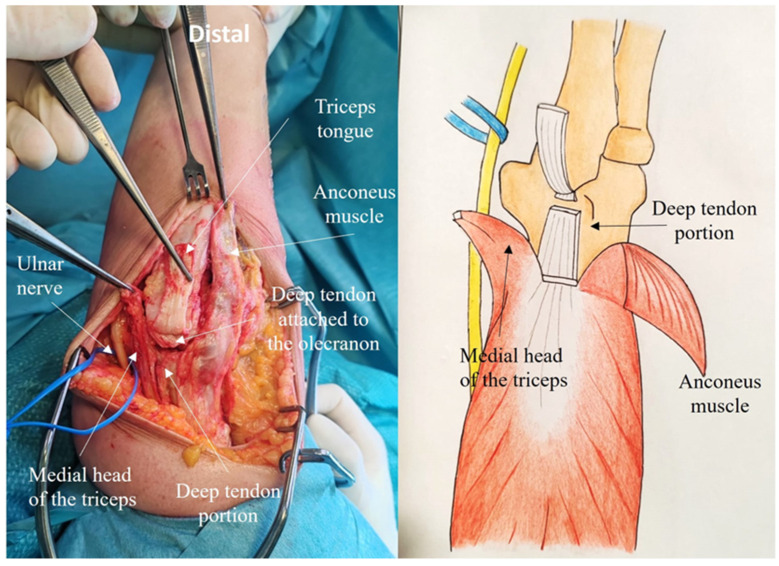
The deep tendinous and muscular portions, originating from the medial head of the triceps, are incised longitudinally and reflected proximally together with the anconeus flap. The posterior capsule is exposed and elevated. The olecranon fossa is debrided.

The deep tendon and muscle portions, originating from the medial head of the triceps, are incised longitudinally and reflected proximally together with the anconeus flap, leaving 1 cm of the deep tendon attached to the olecranon (Figure 6). The posterior capsule is exposed and elevated, the olecranon fossa is debrided.

Step 2: Joint Exposure and Mobilization

**Figure 7:**
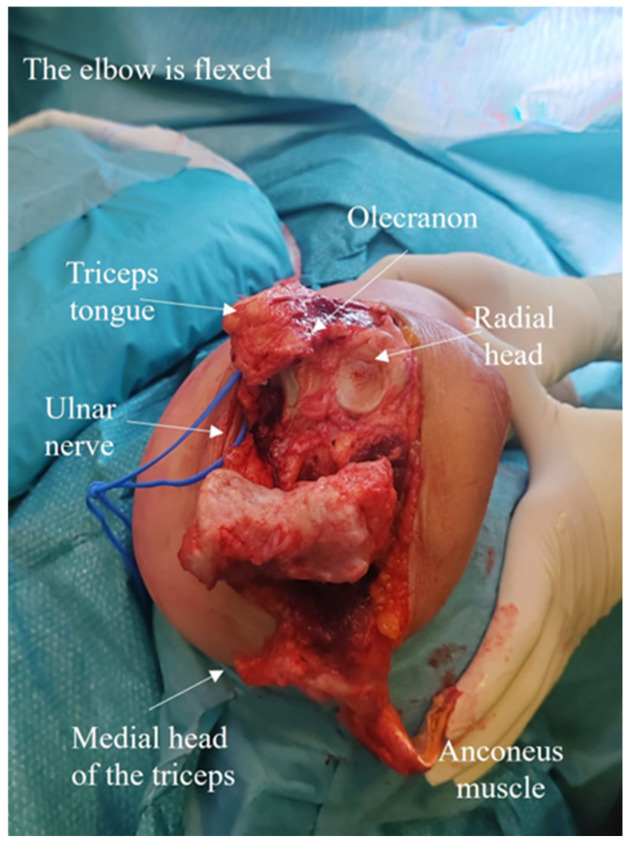
To dislocate the elbow, the shoulder is externally rotated and adducted, the elbow is flexed, and the forearm is in neutral rotation.

Reflecting the anconeus flap with the triceps proximally and the triceps tongue flap distally affords full visualization of the posterior elbow joint and humeral metaphysis/diaphysis. The medial and lateral collateral ligaments are released from the humerus; the flexor-pronator complex must also be detached medially and the extensor complex laterally. The anterior capsule is elevated, directly on the bone, to prevent injury to the neurovascular structures anteriorly. Elbow dislocation toward the ulna provides excellent joint exposure. To dislocate the elbow, the shoulder is externally rotated and adducted and the elbow is flexed with the forearm in neutral rotation (Figure 7).

Step 3: Implant placement

The distal humerus and ulna preparation for the prosthetic components is according to standard techniques. Bone cuts are made under direct visualization, facilitated by the extensive exposure afforded by the ARTT approach. After inserting the trial components, ROM testing allows for evaluating stability, alignment, and component positioning. The final components are implanted and cemented as appropriate.

Step 4: Closure

**Figure 8:**
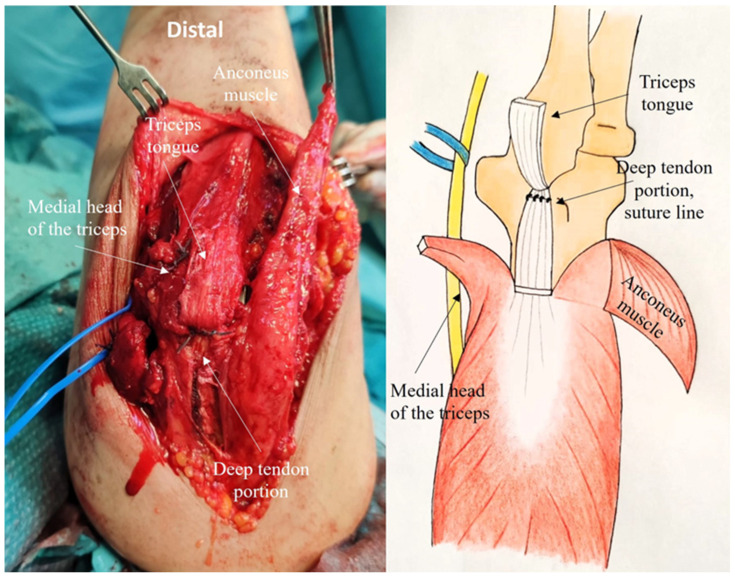
The deep portion of the proper tendon is sutured to the portion of the tendon attached to the olecranon.

Suturing plays a crucial role in tendon healing. It begins by securing the deep portion of the proper tendon to the tendon portion attached to the olecranon (Figure 8).

**Figure 9:**
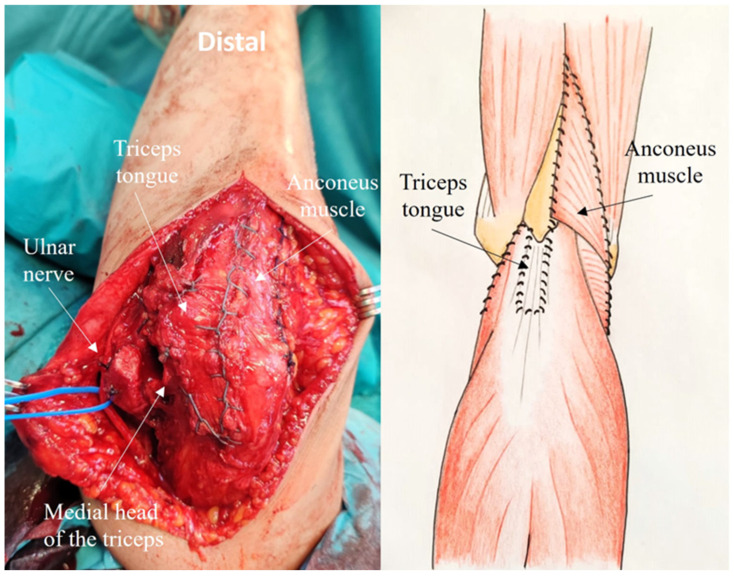
The four corners of the triceps tongue flap are closed with a nonabsorbable suture and a running suture is performed to close the anconeus flap and the medial portion of the triceps.

The four corners of the triceps tongue flap are closed with nonabsorbable sutures; then, a running suture is performed to close the anconeus flap and the medial portion of the triceps (Figure 9).

Sutures should be tied with the elbow in 60° of flexion. After transposing the ulnar nerve into subcutaneous tissue, an absorbable running suture is performed to close the subcutaneous pocket. Three drains are inserted and kept for the following 24 h. The skin is closed and the arm is placed in a rest splint at 90° of flexion for 24 h.

### 2.5. Postoperative Program

The splint is worn for the first 24 h. Passive motion in flexion–extension and pronation–supination is allowed from the next day, even with the drains in place. Gentle passive and active motions are begun without triceps protection and progress as a home program, increasing to full ROM over 2–4 weeks. Patients are advised to avoid lifting objects weighing more than 4.5 kg and to avoid lifting repetitively any object weighing more than 2 kg with the injured arm. After surgery, prophylactic therapy for heterotopic ossifications with non-steroidal anti-inflammatory drugs (e.g., celecoxib 200 mg) was performed for 21 days in all the patients.

### 2.6. Statistical Analysis

The demographic data are described using percentages. The variables (ROM, pain, triceps function, MEPS, VAS) are reported as mean and standard deviation (s.d.).

We tested the normal distribution of data with the Shapiro–Wilk test, calculated the standard deviation (s.d.), and evaluated homoscedasticity with the F test for homogeneity of variances. Afterwards, we performed Student’s t-test to compare the outcomes of two independent groups. Percentages of qualitative dichotomic variables were analyzed with the Fisher exact test. The level of significance was set at *p* < 0.05 in all the tests, that were 2 tailed. We reported data as mean (range, minimum value–maximum value). Analysis was performed using the XLSTAT(2024.3) software for Microsoft Office Excel^®^.

## 3. Results

All patients were available for clinical and radiographic follow-up until at least 24 months (mean, 29; range 26–32; Table 2).

### 3.1. Clinical Outcomes

The MEPS increased from 39 (range, 25–55, s.d., 11.5) before the procedure to 95 points (range 85–100, s.d., 7.5) at the time of the last evaluation (*p* value < 0.01). The VAS score for pain fell from 7.5 (range 6–9; s.d., 7.5) to 1 (range, 0–3, s.d., 1), respectively (Table 2). The preoperative arc of motion in flexion–extension was 48° (range, 30–80; s.d., 18) and began at 11° (range, 0–30; s.d., 12), with flexion at 60° (range, 50–90; s.d., 20). At the time of the last evaluation, the flexion–extension arc was 113° (range, 110–130; s.d., 8.1, *p* value < 0.01) and began at 20° (range, 10–30; s.d., 8.9) with flexion at 133° (range, 120–140; s.d., 8.1). The mean increase in the arc of motion in flexion extension was 90°. Before the procedure, the mean arc of forearm rotation was 126° (range, 40–180; s.d., 55), whereas after the procedure it was 175° (range, 150–180; s.d., 12.2, *p* value = 0.062). All six patients were able to hold the elbow against gravity with the forearm extended over the head, like the contralateral arm. Five patients achieved the same triceps strength as the contralateral arm and grade 5 on the MRC scale [27]. The sixth patient recovered triceps function against resistance over the entire ROM, though the arm was weaker than the contralateral side, and achieved grade 4. One patient had ulnar neuropathy, which was resolved by the 6th week.

### 3.2. Radiographic Outcomes

**Figure 10:**
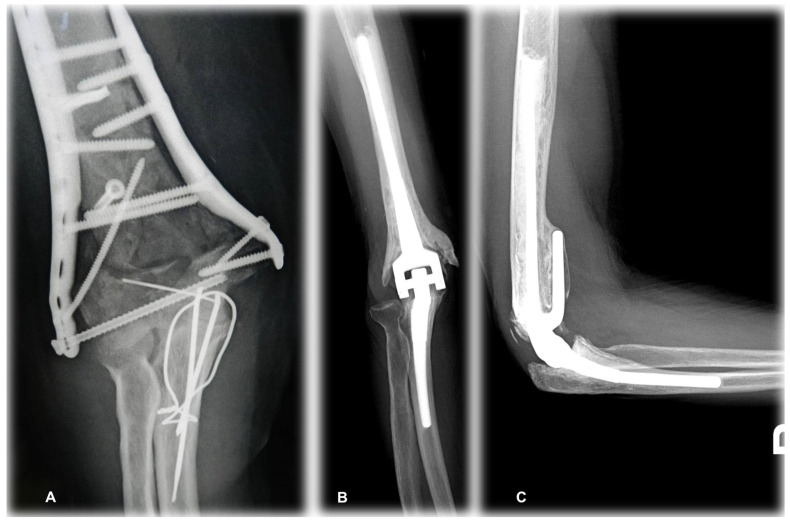
Preoperative (**A**) and Postoperative (**B**,**C**) X-rays (Case 4).

A review of the postoperative radiographs showed inadequate cement distribution in one elbow, due to the fact that the cement had not reached the tip of the ulnar and of the humeral components. The mean radiological follow-up was 29 months (range, 26–32), similar to the clinical follow-up, and showed no evidence of component loosening. Radiolucent lines [17] (type 1) were detected in one elbow. Heterotopic ossification was observed in 2 patients (cases 2 and 6), who were class IIA according to Hastings and Graham [28]. There were no signs of bushing wear at the time of the last radiographic assessment. None of the implants required revision. (Figure 10)

## 4. Discussion

In the past decade, TEA has proved to be an increasingly useful procedure, enhancing ROM and relieving pain in a growing number of patients with a variety of trauma-related elbow conditions. In several countries, TEA is the most common primary elbow replacement procedure [29,30]. In individuals with severe elbow dysfunction, such as post-trauma patients, TEA typically involves a more difficult surgical exposure, due to bone joint deformity and scar tissue formation around the entire joint. In particular, tissue scarring and triceps tendon retraction seem to be more severe in patients whose previous procedures have affected the triceps tendon-bone interface. Posterior surgical approaches are the most widely used for TEA, as they afford excellent exposure to the articular surface, which enhances outcomes. Numerous approaches with different types of triceps exposures are available for TEA. In all cases, it is essential to preserve the olecranon bone structure to avoid compromising the fixation of the ulnar component [2,9,12,31,32,33]. Morrey et al. [5] have described three options to manage the triceps mechanism: (i) leaving the triceps tendon attached to the olecranon, either by stripping the distal humerus or by turning down the aponeurosis [5]; (ii) reflecting it in continuity with soft tissue, with/without a portion of the bone attachment [2,32,34]; and (iii) splitting it along the midline [5,13]. A further key element in joint exposure for TEA is the role of the anconeus muscle in the extensor mechanism. According to Basmajian and Griffin [35], who have provided a highly accurate description of its function, the main role of the anconeus is to stabilize the joint in extension and against resistance, though it may also stabilize the ulna during forearm hypersupination. This view has recently been confirmed by electromyography studies, which have detected simultaneous contractions of the lateral side of the triceps and anconeus muscles. These findings lend further support to the notion that the synergistic relationships between muscles are dynamically dependent on the functions of the extensor apparatus [36,37,38]. Selection by the surgeon of the triceps approach can also be based on the severity of the condition, personal preference, and/or the exposure of the ulnohumeral joint required to optimize component implantation, particularly in post-trauma patients. Extensor mechanism insufficiency is a well-recognized complication of TEA [3,39]. In post-trauma patients, its incidence ranges from 1% to 29% [11,19,22,23,24], but is probably underreported, and is higher in those patients where the triceps tendon insertion has been violated by previous procedures or damaged by the trauma.

Extensor apparatus insufficiency is related to poor tissue quality or to failed surgical reinsertion before healing completion, particularly in elbows managed by triceps takedown approaches or affected by a new trauma episode [3,39]. Patients with triceps weakness can actively extend the elbow against gravity, but not against resistance. Extensor weakness may also be related to biological factors, such as a scant quality of the original tendon or poor tendon healing in elbows where the surgical approach has violated the tendon-bone interface. Mechanical factors may also contribute, such as changes in the triceps moment arms secondary to an incorrect position of the prosthetic hinge involving changes in the rotational axis as a consequence of inadequate component positioning [4,10,18,20,40,41]. Component malalignment contributes to increased forces through the linked system, inducing polyethylene edge loading and increased torsion at the implant-bone interface and at the tips of the prosthetic components. The hinge mechanism and bushing system appear to be the elements at the highest risk of failure [8,21,42,43]. A different condition is triceps insufficiency due to secondary avulsion of the tendon from the ulna, which manifests as loss of extension strength as well against gravity and inability to perform overhead activities. A biomechanical test study by Petre et al. [20] has found that triceps tendon rupture occurred at the tendon-bone interface, proceeding laterally from the medial olecranon insertion. Other clinical and surgical investigations have described lateral dislocation of the triceps tendon in continuity with the forearm fascia, periosteum, and anconeus occurring from medial to lateral [3,39]. In contrast, no cases of triceps insufficiency have been described in triceps-on approaches [1,15,39,44]. The triceps sparing or para-tricipital approaches are alternative exposures for TEA that do not entail triceps takedown and do not affect the triceps tendon insertion on the olecranon. In a study comparing their results with triceps-reflecting and triceps-sparing approaches, Pierce and Herndon [15] observed a high incidence (60%) of triceps weakness in the former group and no weakness or insufficiency in the latter.

The disadvantages of preserving the whole triceps are the risk of inadequate olecranon exposure and of more complicated ulnar canal preparation and component positioning. Also, there is a risk of damage to the triceps tendon insertion when trying to extend the exposure and of inadequate rotation of the ulnar component, particularly in post-trauma patients, where bone deformity and tendon retraction often hamper joint exposure.

Based on the above considerations, we have devised the ARTT approach, which we feel combines optimal joint exposure and preservation of the tendon insertion on the olecranon, particularly in post-trauma patients with severe elbow dysfunction [2,9,12,31,32,33]. In this approach, reflecting the triceps muscle and the anconeus as one on the lateral portion of the triceps spares the olecranon insertion of the triceps tendon. This optimizes joint visualization and exposure of the olecranon and the humerus and allows for the performance of all the surgical steps without traction on the tendon-bone insertion, thus reducing the risk of secondary tendon rupture or attenuation. The ARTT approach is a triceps-sparing approach based on the anatomical differences between the lateral and medial sides of the tendon. In fact, the lateral part is larger and relatively thin and is continuous with the anconeus muscle and the fascia, whereas the medial portion is thicker, like a proper triceps tendon. Medially, the combined triceps tendon (medial, lateral, and long heads) inserts directly onto the medial-most part of the posterior aspect of the olecranon [6,7,45,46,47]. Although the three heads are combined, the deep medial head footprint is separate from the superficial lateral and long heads. The common insertion is called the proper tendon insertion, whose mean width and thickness are about 20 mm and 8 mm, respectively. The anconeus muscle as part of the extensor mechanism complex originates from the posterior aspect of the lateral epicondyle and from the lateral triceps fascia. It inserts on the radial aspect of the olecranon and proximal ulna. Its innervation is supplied from the terminal branch of the radial nerve and runs along the lateral portion of the medial head. Its blood supply comes from the interosseous recurrent artery [6,46,47]. The ARTT approach ensures the preservation of the anconeus muscle in continuity with the triceps and of its nerve and blood supplies.

The short-term outcomes of our six patients suggest that the ARTT approach combines the advantages of triceps-splitting and triceps-reflecting approaches, i.e., adequate joint exposure and a lower risk of triceps insufficiency, and of triceps-sparing approaches, i.e., the integrity of the triceps olecranon insertion. Healing occurs at the muscle-tendon surface, not at the bone-tendon interface. This is also the reason why at two years patients reached stages 4 or 5 of the MRS scale [26,27] and were able to extend the forearm against gravity.

This triceps management procedure also affects the rehabilitation program. Patients undergoing detachment of the tendon insertion from the olecranon must avoid active elbow extension for 4–6 weeks, to protect the healing of the tendon reinsertion. In contrast, sparing of the tendon-bone insertion in the ARTT approach enables early passive and active mobilization in the full ROM and only requires avoiding extension against gravity in the first two weeks. In these patients, pain at rest or during rehabilitation is often merely a tolerable discomfort secondary to the tissue response to surgery. Notably, the VAS pain score [25] fell from 7.5 before the procedure to one at 24 months.

The ARTT approach is not without disadvantages. Its chief limitation is its technical difficulty, which demands a thorough understanding of anconeus-triceps anatomy. In particular, careful tissue dissection to avoid damaging the radial nerve or blood vessels, the steps involved in reflecting the anconeus and triceps muscles as one, and preparation of the triceps tongue while preserving the deep tendon may involve a learning curve for the less experienced surgeons.

## 5. Conclusions

In conclusion, the ARTT approach offers a viable alternative to current TEA procedures, particularly in post-trauma patients, who benefit from improved joint access as they often present scarring, hardware problems, bone deformity, and/or significant joint destruction. Its chief limitation is its technical difficulty, which demands a thorough understanding of anconeus-triceps anatomy. The small sample size of this report needs further investigations to validate its effectiveness and assess potential long-term complications, related to the ARTT approach and to the TEA. We are collecting more cases to assess and compare the outcomes with other triceps approaches used for TEA in post-traumatic sequelae, with several previous surgical exposures.

## Figures and Tables

**Table 1:** Preoperative demographic and clinical data of the six patients.

Case	Gender	Age	Side	Extension	Flexion	F-E Arc	Pronation	Supination	P-S Arc	Pain (MEPS)	Stability (MEPS)	Motion (MEPS)	Activities (MEPS)	Total MEPS	Previous Procedures (no.)	VAS
1	F	79	L	0°	40°	40°	20°	20°	40°	15	0	5	5	25	2	6
2	F	73	R	30°	70°	40°	40°	80°	120°	15	5	5	5	30	3	9
3	F	68	R	20°	50°	30°	90°	90°	180°	15	5	5	15	40	2	7
4	F	66	R	0°	40°	40°	40°	50°	90°	15	10	5	5	35	2	7
5	M	57	L	10°	70°	60°	60°	90°	150°	15	10	15	10	50	4	8
6	M	63	R	10°	90°	80°	90°	90°	180°	15	10	15	15	55	2	8

**Table 2:** Postoperative clinical data of the six patients.

Case	Follow-up (Months)	Extension	Flexion	F-E Arc	Pronation	Supination	P-S Arc	Pain (MEPS)	Stability (MEPS)	Motion (MEPS)	Activities (MEPS)	Total MEPS	Triceps function Mrc Scale	VAS
1	29	10°	140°	130°	90°	90°	180°	45	10	20	25	100	5	0
2	28	20°	130°	110°	90°	90°	180°	30	10	20	25	85	5	3
3	32	10°	120°	110°	90°	90°	180°	45	10	20	25	100	5	0
4	31	30°	140°	110°	80°	70°	150°	45	10	20	25	100	5	0
5	30	20°	130°	110°	90°	90°	180°	30	10	20	25	85	4	3
6	26	30°	140°	110°	90°	90°	180°	45	10	20	25	100	5	0

## Data Availability

Dataset available on request from the authors.

## Abbreviations

The following abbreviations are used in this manuscript:

TEA	Total Elbow Arthroplasty
ARTT	Anconeus-reflected Triceps tongue
ROM	Range of Motion
RL	Radiolucent lines
F-E	Flexion–Extension
P-S	Pronation–Supination
MEPS	Mayo Elbow Performance Score
MRC Scale	Medical Resource Council Scale as modified by Paternostro-Sluga
VAS	Visual Analog Scale
